# “Sentinel Oculomotor Nerve Palsy”: A Harbinger of Subarachnoid Hemorrhage

**DOI:** 10.1155/crnm/6786272

**Published:** 2025-05-13

**Authors:** Vijay Renga

**Affiliations:** Department of Neurology, Medical University of South Carolina, Charleston, South Carolina, USA

## Abstract

Subarachnoid hemorrhage (SAH) is a life-threatening condition most commonly caused by aneurysmal rupture. Sentinel headaches, often described as the “worst headache of life” or a “thunderclap headache,” are critical warning signs that may precede SAH. However, atypical headaches can complicate early diagnosis. Oculomotor nerve palsy, though rare, may occur as a complication of both aneurysmal and nonaneurysmal SAH. We report a unique case of a 64-year-old woman who initially presented with atypical headache followed by isolated oculomotor nerve palsy, preceding the onset of a nonaneurysmal SAH. This case highlights isolated oculomotor palsy as a potential unrecognized sentinel sign of SAH.

## 1. Introduction

Subarachnoid hemorrhage (SAH) is a medical emergency characterized by bleeding into the space between the arachnoid membrane and the pia mater surrounding the brain. Approximately 80% of nontraumatic cases are due to ruptured intracranial aneurysms. Other etiologies include arteriovenous malformations, cerebral amyloid angiopathy, vasculitis, and reversible cerebral vasoconstriction syndrome. Patients with SAH typically present with a sudden, severe headache—often described as the worst headache of their life—accompanied by nausea, vomiting, neck stiffness, and altered mental status. Prompt neurosurgical intervention is usually required to secure the aneurysm or control the bleeding, along with supportive care to manage complications such as vasospasm and hydrocephalus.

A sentinel or “warning” headache is a sudden, intense headache that may precede aneurysmal rupture or SAH. It is distinguished by its abrupt onset and severe intensity, often described as unlike any previous headache [1]. While abrupt, severe headache is seen in only about half of patients with aneurysmal SAH, it may occur in up to two-thirds of patients with benign thunderclap headache, posing a diagnostic challenge. In SAH, loss of consciousness occurs in approximately 26% of cases, while transient focal neurological symptoms are seen in about 33%. Seizures and diplopia may also occur, albeit less frequently [2].

Isolated oculomotor nerve palsy can result from aneurysmal compression or may occur as a complication of SAH [2, 3]. Here, we present a rare case of a 64-year-old woman who developed isolated oculomotor palsy after experiencing atypical headaches, followed by a nonaneurysmal SAH (NASAH). This case underscores the need to consider oculomotor nerve palsy as a potential premonitory symptom—or ‘sentinel sign'—of SAH, even in the absence of aneurysmal findings.

## 2. Case Presentation

A 64-year-old woman presented to urgent care with complaints of right-sided headache and facial pain that had persisted for three days. She had a remote history of trigeminal neuralgia over 30 years prior. She was referred to the emergency department, where a noncontrast CT scan of the head was performed and found to be normal. She was diagnosed with trigeminal facial pain or possible shingles and was discharged with pain medications, steroids, and valacyclovir.

Four days later, she returned to the hospital after developing an inability to open her right eye. She described a burning pain on the right side of her nose radiating to the back of her head. Her medical history was notable for right-eye glaucoma.

Neurological examination revealed a complete right oculomotor nerve palsy characterized by ptosis, lateral deviation of the eye, and a nonreactive, dilated right pupil. Repeat noncontrast head CT and CT angiography were both negative for acute findings, including aneurysms or vascular abnormalities. Laboratory investigations showed neutrophilic leukocytosis (WBC 12,000/μL), with normal inflammatory markers (ESR 7 mm/hr and CRP < 0.5 mg/L).

The following day, brain MRI was unremarkable. Ophthalmologic evaluation confirmed elevated intraocular pressure (33 mmHg) in the right eye and visual acuity of 20/50. She was prescribed eye drops and scheduled for outpatient follow-up, as no clear etiology for the oculomotor palsy had been identified.

**Figure 1:**
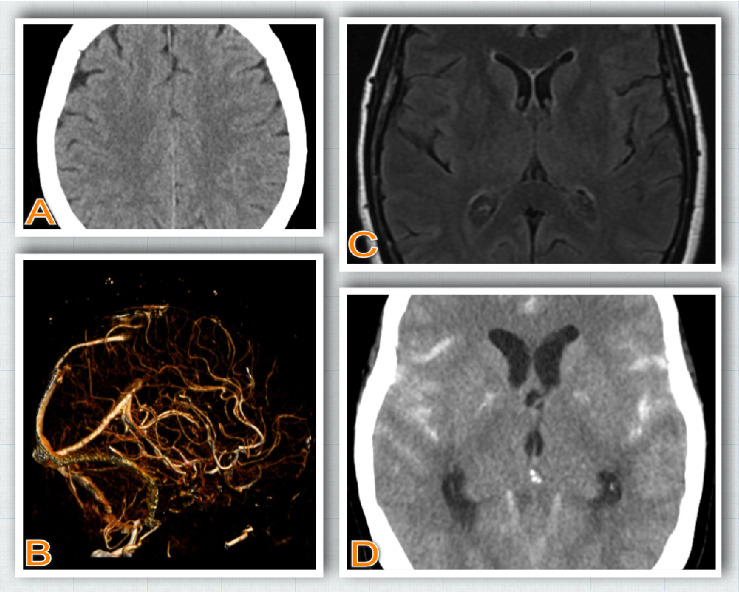
(A) Day 1 CT scan (coronal slice): no hemorrhage. (B) Day 1 CT angiogram (3D reconstruction): no evidence of aneurysm. (C) Day 2 MRI brain: normal. (D) Day 2 CT Head: Diffuse subarachnoid hemorrhage.

Given the diagnostic uncertainty and concern for a possible central nervous system process, a lumbar puncture was performed. Cerebrospinal fluid (CSF) analysis revealed xanthochromia and grossly bloody fluid, raising concern for SAH. A repeat head CT subsequently confirmed diffuse SAH (see Figure 1).

## 3. Discussion

SAH is a life-threatening neurological emergency. Approximately 80% of nontraumatic SAH cases are attributed to aneurysmal rupture, though a minority is classified as NASAH [4]. Other nontraumatic causes include vascular malformations such as arteriovenous malformations and vasculitis, as well as conditions like cerebral amyloid angiopathy and reversible cerebral vasoconstriction syndrome. SAH carries a high mortality rate, with nearly 50% of affected patients dying, and only one-fourth achieving a good recovery without neurological deficits [5].

Oculomotor nerve palsy is a recognized presenting sign of posterior communicating artery aneurysm or aneurysmal SAH. The oculomotor nerve originates from the midbrain and travels through the cavernous sinus before entering the orbit via the superior orbital fissure. It divides into superior and inferior branches, innervating extraocular muscles critical for eye movement and eyelid elevation. Its parasympathetic fibers also innervate the pupillary sphincter, mediating the light reflex. Aneurysms causing oculomotor palsy most commonly occur at the junction of the internal carotid artery and the posterior communicating artery [6]. Due to this close anatomical proximity, compression of the oculomotor nerve can result in symptoms such as ptosis, diplopia, and a dilated, nonreactive pupil—a classic “blown pupil.”

While most commonly associated with posterior communicating artery aneurysms, oculomotor nerve palsy has also been reported in association with aneurysms of the internal carotid, anterior cerebral, posterior cerebral, superior cerebellar, and basilar arteries [7, 8]. In SAH, the nerve may also be affected indirectly due to increased intracranial pressure or blood accumulation in the subarachnoid space [3]. The extent of nerve involvement can vary, from partial (fascicular) palsy with pupillary sparing [9] to complete paralysis, as observed in our patient.

Although rare, oculomotor nerve palsy has been described in the setting of NASAH, and its occurrence in the absence of an aneurysm generally indicates a more favorable prognosis compared to aneurysmal SAH [10]. In our patient, third nerve palsy developed a day prior to radiographic evidence of hemorrhage, following 3 days of warning headaches. This sequence—progression from atypical headache to isolated oculomotor palsy and subsequent NASAH—has not been previously reported.

This case raises important diagnostic considerations. The patient's initial symptoms, including facial pain and a history of trigeminal neuralgia, contributed to diagnostic uncertainty. The CT angiogram was negative, as is sometimes the case, particularly for aneurysms smaller than 3 mm. While CT angiography has a reported sensitivity of over 96% for aneurysms larger than 3 mm [11], it is not definitive. In our case, lumbar puncture proved to be a crucial diagnostic step. CSF analysis confirmed SAH, reinforcing the importance of LP in cases where CT and CTA are inconclusive. Lumbar puncture remains nearly 100% sensitive for detecting SAH, compared to approximately 95% sensitivity with CT alone.

The premonitory headache in this patient, which began nearly a week before the hemorrhage, lacked the classic features typically associated with a sentinel headache, thereby complicating the initial diagnosis. The subsequent development of isolated oculomotor nerve palsy—occurring 4 days prior to the confirmed hemorrhage—appears to represent a previously undocumented sentinel sign of SAH, particularly in the context of nonaneurysmal SAH. Potential underlying mechanisms may include microvascular ischemia or early localized compressive effects from a minor or sentinel bleed preceding the full hemorrhagic event. Early recognition of this clinical pattern may facilitate prompt diagnosis and enable timely, potentially life-saving interventions such as digital subtraction angiography (DSA), even when initial noninvasive imaging is unrevealing.

## 4. Conclusion

Oculomotor nerve palsy can arise from a variety of causes, including aneurysms, vascular malformations, diabetes, and inflammatory or infectious processes. This case emphasizes that third nerve palsy of unclear etiology, especially when accompanied by headache, should prompt consideration of SAH—even in the absence of aneurysmal findings. Recognition of isolated oculomotor palsy as a potential sentinel sign of SAH could enable earlier diagnosis and timely intervention, which may be lifesaving.

## Data Availability

The data are available upon request.
